# Delayed pollination and low availability of assimilates are major factors causing maize kernel abortion

**DOI:** 10.1093/jxb/ery013

**Published:** 2018-02-09

**Authors:** Si Shen, Li Zhang, Xiao-Gui Liang, Xue Zhao, Shan Lin, Ling-Hua Qu, Yun-Peng Liu, Zhen Gao, Yong-Ling Ruan, Shun-Li Zhou

**Affiliations:** 1 College of Agronomy and Biotechnology, China Agricultural University, Beijing, China; 2 School of Environmental and Life Sciences, The University of Newcastle, Callaghan, NSW, Australia; 3 Scientific Observation and Experimental Station of Crop High Efficient Use of Water in Wuqiao, Ministry of Agriculture, Wuqiao, China

**Keywords:** Abortion, thylene, ructose, lucose, invertase, kernel set, maize, pollination time, sucrose

## Abstract

Selective seed abortion is a survival strategy adopted by many species that sacrifices some seeds to allow the remaining ones to set. While in evolutionary terms this is a successful approach, it causes huge losses to crop yields. A pollination time gap (PTG) has been suggested to be associated with position-related grain abortion. To test this hypothesis, we developed a novel approach to alter the natural pattern of maize (*Zea mays* L.) pollination and to examine the impact of PTGs on kernel growth and the underlying physiological basis. When apical and basal kernels were synchronously pollinated, the basal kernels set and matured but the apical kernels were aborted at an early stage. Delaying pollination to the basal ovaries suppressed their development and reduced invertase activity and sugar levels, which allowed the apical kernels to set and grow normally. *In situ* localization revealed normal cell wall invertase activity in apical and basal kernels under synchronous pollination but reduced activity in the delayed-pollinated kernels independent of their position. Starch, which was abundant in basal kernel areas, was absent in the apical kernel regions under synchronous pollination but apparent with delayed pollination. Our analyses identified PTG-related sink strength and a low level of local assimilates as the main causes of grain abortion.

## Teaser

Manually delaying pollination in maize restricted kernel growth and induced abortion by suppressing cell wall invertase activity and repartitioning assimilates.

## Introduction

The angiosperms are known to produce more flowers and ovules than mature fruits and seeds. Competition for survival among developing progenies results in selective fruit and seed abortion to allow the remaining ones to survive (Stephenson, 1981; Ganeshaiah and Shaanker, 1988; Ruan, 2014; Tardieu *et al.*, 2014). Ovule abortion occasionally occurs during the pre-fertilization stage owing to (i) silk senescence (Bassetti and Westgate, 1993; Bassetti and Westgate, 1994) or (ii) insufficient or feeble pollen grains (Campbell and Halama, 1993; Ehrlen and Eriksson, 1995; Goldingay, 2000). However, pollen limitation is not a common occurrence in many angiosperms (Bawa and Webb, 1984; Burd, 1994; Larson and Barrett, 2000; Sakai and Harada, 2001). Instead, post-fertilization seed abortion can frequently be induced due to limitations in the available resources (Snow and Spira, 1991; Arathi *et al.*, 1999; Yang *et al.*, 2005; Brookes *et al.*, 2008; Arathi, 2011), and it can become more severe under unfavorable circumstances, including drought (Otegui *et al.*, 1995; Andersen *et al.*, 2002; McLaughlin and Boyer, 2004a, b), heat (Cheikh and Jones, 1994; Hays *et al.*, 2007; Liu *et al* 2016), and other environmental stresses (Andrade *et al.*, 2002; Hiyane *et al.*, 2010). Given the increased frequency and severity of climate-change-related stresses, seed abortion has become a central issue for food security in the 21st century (Grassini *et al.*, 2013; Ray *et al.*, 2013).

Maize (*Zea mays* L.) is one of the most important crops in the world, ranking third in production acreage and first in grain production worldwide. In practical production, the maize ear shows a base-to-apex gradient of abortion frequency, in that kernels in the apical area have a higher probability of abortion (Cárcova and Otegui, 2001; Uribelarrea *et al.*, 2008; Feng *et al.*, 2011). The phenomenon of non-random kernel abortion is similar to that in those species with a linear arrangement of seeds, such as legumes (Rocha and Stephenson, 1990, 1991), wheat (Yang *et al.*, 2006; Hays *et al.*, 2007), and rice (Wang *et al.*, 2011; Chen *et al.*, 2013). In these species, the probability of seed abortion is highly related to the seed position in the ear or panicle. However, seeds from the disadvantageous position can develop normally on culture medium containing sufficient nutrients (Nakamura, 1986), or if some of the seeds from the more advantageous position are removed (Rocha and Stephenson, 1991; Feng *et al.*, 2011). These observations indicate that the linear probability of abortion is not due to poor seed vigor. Instead, position-related abortion may relate to poor access to maternal resources (Watson and Casper, 1984) and the time of fertilization (Cárcova and Otegui, 2001). However, there is still a lack of experimental evidence to support these theories, despite the existence of a large number of studies investigating the possible basis of seed abortion at the physiological and molecular levels, including sugar deprivation (Hiyane *et al.*, 2010; Kakumanu *et al.*, 2012), ethylene regulation (Hays *et al.*, 2007; Feng *et al.*, 2011), and deficiency in invertase activity (Ruan *et al.*, 2012). In this context, the pollination time gap (PTG) was recently suggested to be an initial and primary cause of maize kernel abortion, which occurred earlier than abortion due to sugar depletion in response to drought (Oury *et al.*, 2016a, b). Whether these putative factors are the causes or the consequences of seed abortion is still disputed, and little is known about their relationships with PTGs in linear-arranged seed or grain abortion (Boyer and McLaughlin, 2007; Ruan *et al.*, 2012; Oury *et al.*, 2016*a*). We aimed to address these issues by using the maize kernel as an experimental model.

Technically, it is extremely difficult to appreciate the effects of PTGs on maize kernel growth. PTGs depend on the time differences between the pollination of early- and late-appearing silks. The pattern is difficult to change because maize silks emerge and elongate under the protection of husks. Some previous studies attempted to change the PTGs by altering growth conditions via heat or water deprivation treatments during the silking period (Cárcova and Otegui, 2001; Oury *et al.*, 2016a, b). However, altering growth conditions influences not only the PTGs, but also kernel growth and metabolism. In this study, we tested the effect of PTGs on maize kernel development by using an innovative approach in which we cut the basal silks to prevent natural pollination and then provided them with fresh pollen grains at the specified time points to conduct delayed pollination (DP) of the basal kernels. In this way, the inherent pollination pattern was changed without altering the environmental conditions. The effects of PTGs on kernel growth, invertase activity, and sugar and ethylene levels were investigated.

## Materials and methods

### Plant materials and management

Maize plants were grown at the Wuqiao experimental station of China Agricultural University (37°29ʹ–37°47ʹN; 116°19ʹ–116°42ʹE) in Hebei Province, China. Two maize hybrids, *ZhengDan 958* (*ZD958*) and *DengHai 605* (*DH605*), with normal and large ear sizes, respectively, were chosen for use in the experiments. Seeds were sown at a density of 90000 plants/ha with basal compound fertilizer (750 kg/ha; N 15%, P_2_O_5_ 15%, K_2_O 15%) and top-dressed with urea (245 kg/ha; N 46%) applications at the 13-leaf stage. The ears of chosen plants were bagged before the silking stage and then were hand-pollinated. Pesticides were applied as necessary to protect the plants from insects and diseases. There was no evidence of either water or nutrient stress during the growth period.

### Identification of basal silks, basal kernel delayed pollination, and validation of the approach

To verify the location of basal silks among the silk cluster, we carefully pulled off husks to observe the spatial structure of silks. As the silks were arranged in an orderly fashion, the silks from the basal area were found to locate at the outer rings of the silk cluster (Supplementary Fig. S1 at *JXB* online). To further verify that the silks were derived from the basal position of the cobs, we cut the silk cluster to a length of 2 cm from the husks at 5 days after the emergence of all silks, so that the silks could be uniformly and neatly arranged. We used a red dye to mark the silks located in the outer rings of the cluster, and then carefully pulled off the husks without breaking off the silks. The positions from which the marked silks initiated on the ear were observed and identified (Supplementary Fig. S1).

**Fig. 1.**
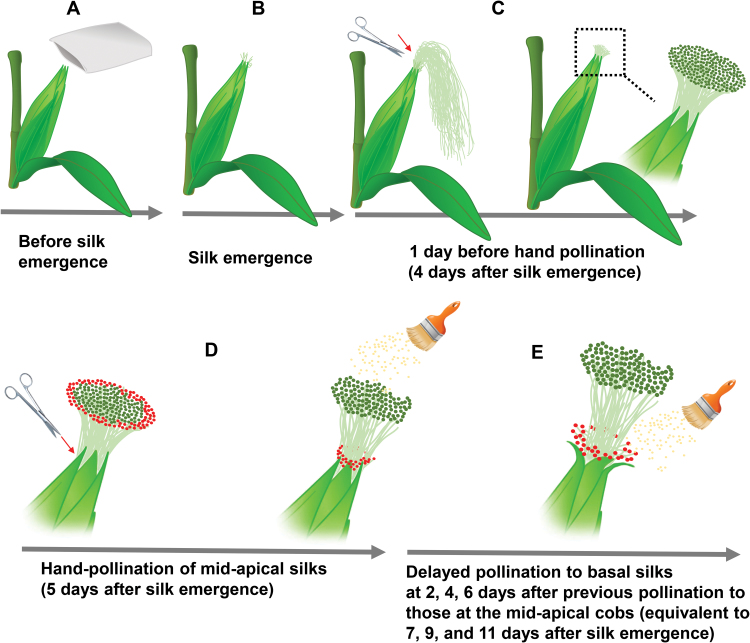
Process of delayed pollination of basal kernels. (A) Before the silks emerged, the selected cobs were covered with paper bags to prevent them from being pollinated naturally. (B) Silks emerged from the middle region of the cobs at ~3 days after being covered. (C) Silks emerged at ~4 days later. Because the silks elongated further and became twisted, making it difficult to pollinate silks derived from different cob positions, we cut the silks at 2–3 cm from the husks, rendering them neatly arranged for the subsequent pollination experiments. (D) Hand-pollination was applied to the silks derived from the mid-apical region of the cobs the next morning. To facilitate this, the basal silks (red) in the peripheral area were cut along the edges of the husks; they became covered by the surrounding husks, thereby preventing them from being pollinated by dropped pollen. Furthermore, the sap secreted from the clipped silks may cause any pollen accidentally dropped on to them to burst and be inactivated. (E) Hand-pollination was applied to the silks developed from the basal region of the cobs 2, 4, and 6 days after the first hand-pollination. At this point, the previously pollinated silks derived from the mid-apical cob region had become withered. However, the majority of the unpollinated basal silks remained receptive to pollen (see Supplementary Fig. S4).

**Fig. 2.**
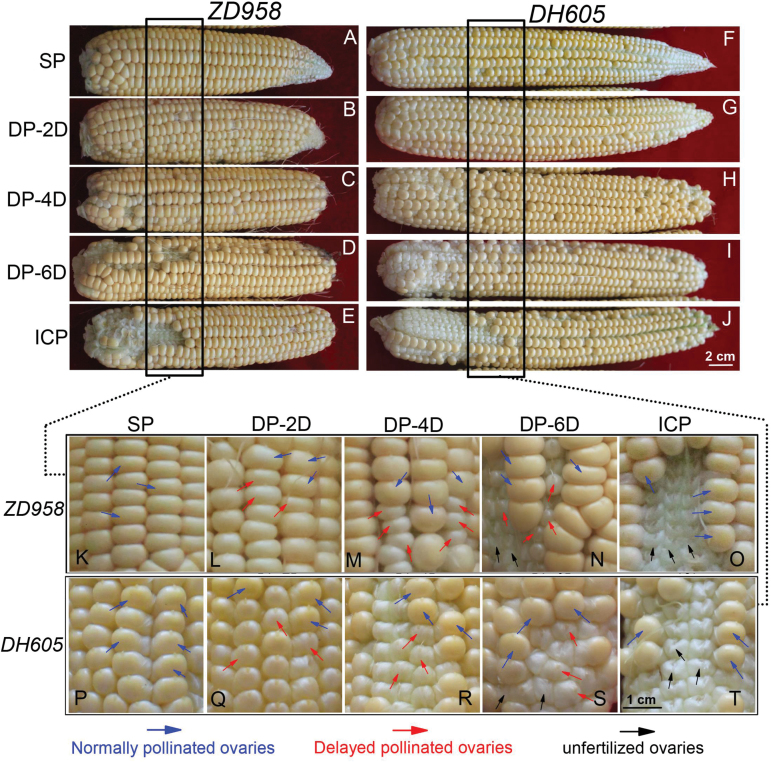
Representative images of maize ears and kernels treated by synchronous pollination (SP), delayed pollination of basal kernels for 2 days (DP-2D), 4 days (DP-4D), or 6 days (DP-6D), and incomplete pollination (ICP). Images were taken at 12 days after pollination of mid-apical kernels. (A–E) Maize hybrid *ZhengDan 958* (*ZD958*) ears; (F–J) *DengHai 605* (*DH605*) ears. The black boxes show the junctions of normally pollinated middle kernels and asynchronously pollinated basal kernels (synchronous pollination in SP, delayed pollination in DP-2D, DP-4D, and DP-6D, and no pollination in ICP). (K–T) Magnified images of the area delineated by the black boxes in A–J. Early pollinated and delayed pollinated kernels in this junction area can be distinguished by color and size. Compared with non-pollinated ovaries in the ICP (negative control) condition, the delayed-pollinated basal kernels are fertilized and visually expanded, but are smaller and lighter in color compared with the basal kernels in the SP treatment (positive control). Blue arrows indicate the middle kernels that were normally pollinated; red arrows indicate the basal kernels that were delayed pollinated in the DP treatments; black arrows indicate unfertilized ovaries.

After identifying the basal silk position, we applied the DP treatment to the basal kernels (Fig. 1; Supplementary Fig. S2). At the junction of the middle and basal kernels, early pollinated and delayed pollinated kernels in the same ear could be easily distinguished by their color and size at an early stage (Fig. 2) or by their size and shape at

maturity (Supplementary Fig. S3). Synchronously pollinated kernels (with normal basal kernels) were used as a positive control, and incompletely pollinated kernels as a negative control (unfertilized ovaries were covered by the glumes; see Fig. 2 and Supplementary Fig. S3).

**Fig. 3.**
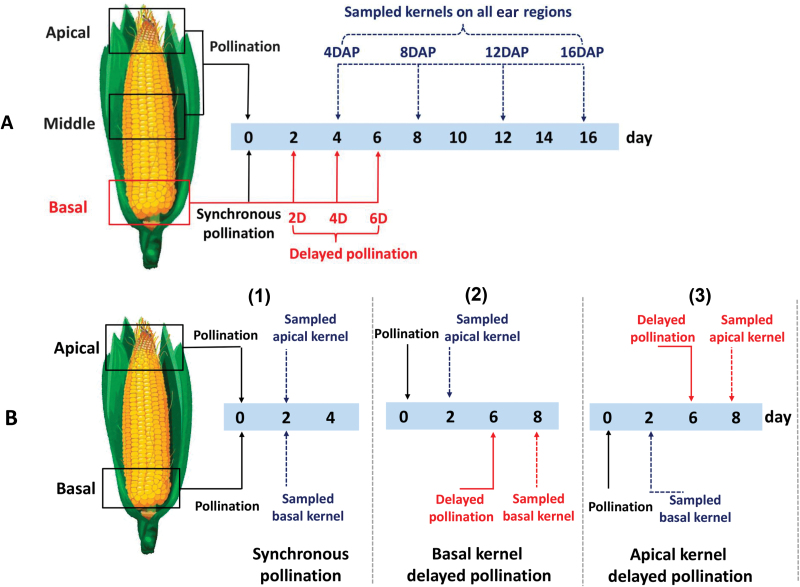
Schematic representation of the two experiments applied to study the effects of pollination time gaps and competition between kernels on different regions of the ear. (A) Experiment I. Black and red arrows indicate the pollination dates for kernels on different regions of the ear in each treatment; blue arrows indicate the dates of sampling for kernels on the apical, middle, and basal ear regions. DAP, days after pollination. (B) Experiment II, designed to demonstrate the effect of delayed pollination on cell wall invertase activity. (1) Synchronous pollination of apical and basal kernels; (2) 6-day-delayed pollination of basal kernels; (3) 6-day-delayed pollination of apical kernels. Black arrows indicate the dates of applying normal pollination; blue arrows indicate the dates of sampling of normally pollinated kernels; red arrows indicate the dates of applying delayed pollination and sampling of the delayed-pollinated kernels.

To assess the level of silk receptiveness to DP, we conducted further experiments to expose emerged silks to fresh pollen grains from the day of silk emergence to 14 days thereafter at 1-day intervals (Supplementary Fig. S4). These experiments revealed that the number of fertilized basal kernels remained unaffected until 9 days after silk emergence, indicating the receptiveness of these silks to DP (Supplementary Fig. S4). Even with pollination at 11 days after silk emergence, we still observed at least 50% of grain set at the basal region, demonstrating the receptiveness of the basal silks at this stage, which is equivalent to pollination delayed by 6 days (see Figs 1 and 3).

### Pollination treatments and sampling

First, an experiment (experiment I) was designed to demonstrate the relationship between pollination and kernel growth in different ear regions during the early growth period (Fig. 3A). Five different pollination treatments were used: synchronous pollination (SP), in which all silks were hand-pollinated synchronously; incomplete pollination (ICP), in which the basal silks were prevented from being pollinated; and three DP treatments, in which the pollination of basal kernels was delayed for 2 (DP-2D), 4 (DP-4D), and 6 days (DP-6D), respectively (Fig. 3A).

Sampling was performed at 4, 8, 12, and 16 days after the first pollination. Apical kernels and all middle kernels were sampled from five rings of kernels on the apical and middle ear regions; the delayed-pollinated basal kernels were identified and sampled. Specifically, the SP apical kernels were chosen from those apical kernels whose growth ceased and were aborted from 4 to 16 days after pollination (DAP). In the DP-6D treatment, owing to the extensive failure of fertilization, only the delayed-pollinated and fertilized kernels were sampled (Fig. 2; Supplementary Fig. S3). The delayed-pollinated basal kernels were identified based on their size and color or shape (as described above). Four biological replicates were used in each case.

A second experiment (experiment II) was conducted to investigate the effect of PTGs on cell wall invertase (CWIN) activity. In this experiment, the *DH605* hybrid was exposed to the SP treatment and basal and apical kernel DP treatments, in which pollination was delayed for 6 days for the basal and apical kernels separately. Fresh kernels were sampled 2 days after the apical or basal kernels were pollinated (Fig. 3B) and were sliced to localize CWIN activity as described by McLaughlin and Boyer (2004*a*) (described below); at this time point, there was no obvious difference in kernel weight or size between apical or basal DP kernels and SP kernels from the same region of the ear.

### Determination of fresh and dry weights

The fresh weight of kernels was measured when they were detached from the ear on the sampling day. The dry weight of the kernel sample was determined after drying the samples at 80 ºC for 48 h to a constant weight.

### Assay of sucrose, glucose, and fructose levels

Carbohydrate extraction was performed according to Hanft and Jones (1986) with slight modifications. Sucrose, glucose, and fructose were then separated using high-performance liquid chromatography (HPLC). Before injection, the carbohydrate extraction solution was filtered through a 0.22 μm Millipore filter. The HPLC system consisted of a Waters 2414 Refractive Index Detector, a Waters 600 Pump, a Waters 600 Controller, and Waters XBridy Amide Columns. The mobile phase was 80% acetonitrile and 20% ultrapure water (containing 0.1% ammonium hydroxide); the pump was set to a flow rate of 1.0 ml min^–1^. The sugar concentrations were quantified using external standards of sucrose, glucose, and fructose (Sigma-Aldrich).

### Starch determination and localization

Starch was extracted according to Hanft and Jones (1986). Starch localization was performed for freshly detached kernels at 8 DAP. The kernels were sliced by hand and then stained with I_2_-KI solution [0.2% I_2_ (w/w) and 0.53% KI (w/w)] for 1 min, briefly washed with water, and viewed under a dissecting microscope.

### Measurement of invertase activity

Cell wall and soluble acid invertase extracts were prepared according to Zinselmeier *et al.* (1999). Invertase activity was determined at pH 4.8 with a reaction in 100 mM sucrose at 30 °C for 30 min. The reaction was terminated by boiling the samples in water for 5 min. Invertase activity was quantified by using a spectrophotometer (Persee Corporation) at a wavelength of 540 nm after reaction with dinitrosalicylic acid solution at 100 °C for 2 min.

### *In situ* localization of cell wall invertase activity

CWIN activity was localized *in situ* according to the method described in McLaughlin and Boyer (2004*a*). Briefly, kernel slices were washed for 5 h with constant stirring, using flowing water to ensure that the soluble sugars in the slices were removed. The washed slices were exposed to the reaction medium for 30 min at 25 °C. The sections were then washed briefly with water, fixed in a 4% solution of formaldehyde, viewed under a dissecting microscope and imaged with a camera.

### Ethylene measurement

Ethylene was collected and measured according to Cheng and Lur (1996) and Feng *et al.* (2011). A gas chromatograph (Shimadzu GC-17A) with a flame ionization detector and an alumina column was used to measure the ethylene emissions. The column, injector, and detector temperatures were 50, 120, and 120 °C, respectively. The carrier gas was N_2_ with a flow rate of 50 ml min^–1^, the burning gas was H_2_ with a flow rate of 70 ml min^–1^, and the assist gas was air with a flow rate of 500 ml min^–1^.

### Statistical analyses and illustration drawing

All measurements described comprised at least four biological replicates. Statistical analyses were conducted by using Student’s *t*-test. For data presented in bar charts, one-way analysis of variance was conducted by using Duncan’s new multiple range test, with statistical significance accepted at *P*<0.05. Statistical analyses were performed using SPSS Statistics and Excel software. Illustrations were drawn in Origin, Adobe Photoshop, and PowerPoint software.

## Results

### Delayed pollination suppressed kernel development

*ZhengDan 958* (*ZD958*) and *DengHai 605* (*DH605*) maize exhibited similar patterns of early kernel development (Fig. 2). The asynchronous hand-pollination treatments broke the temporal patterns of natural pollination (Fig. 2). Basal kernels derived from DP were smaller in size and lighter in color compared with the preferentially pollinated middle kernels and with synchronously pollinated basal kernels in the SP treatment; this difference in appearance was more profound in the treatments with increased PTGs (Fig. 2K–T). The development of apical kernels was obviously improved by delaying or preventing the pollination of basal filaments (Fig. 2A–D, F–I). Specifically, in the DP-6D treatment, some of the basal kernels failed to develop from the fertilization stage (Fig. 2D, I). This failure in kernel development may be due to decreased silk receptivity when pollination is delayed (Bassetti and Westgate, 1993). However, there were still some delayed-pollinated kernels, which were successfully fertilized, developed, and evidently larger than the unfertilized basal ovaries in the ICP treatment (Fig 2N, O, S, T). At maturity, the fertilized aborted basal kernels were smaller and had abnormal shapes compared with the basal kernels in the SP treatment (Supplementary Fig. S3).

**Fig. 4.**
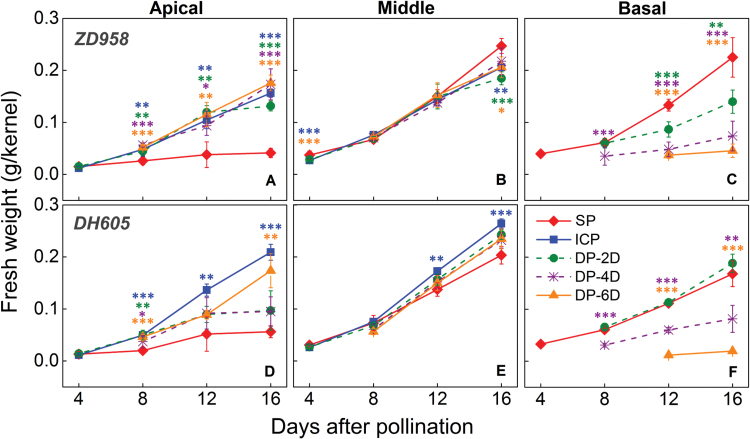
Changes in kernel fresh weight for the maize hybrids *ZhengDan 958* (*ZD958*) and *DengHai 605* (*DH605*) during the early kernel development period. (A–C) Fresh weight of *ZD958* kernels in the apical, middle, and basal ear, respectively. (D–F) Fresh weight of *DH605* kernels in the apical, middle, and basal ear, respectively. DP-2D, pollination time for basal kernels delayed by 2 days; DP-4D, pollination time for basal kernels delayed by 4 days; DP-6D, pollination time for basal kernels delayed by 6 days; ICP, incomplete pollination treatment in which the basal silks were not pollinated; SP, synchronous pollination. Colored asterisks indicate significant differences between the SP and controlled pollination treatments (*t*-test, n=4: *P<0.05, **P<0.01, ***P<0.001).

**Fig. 5.**
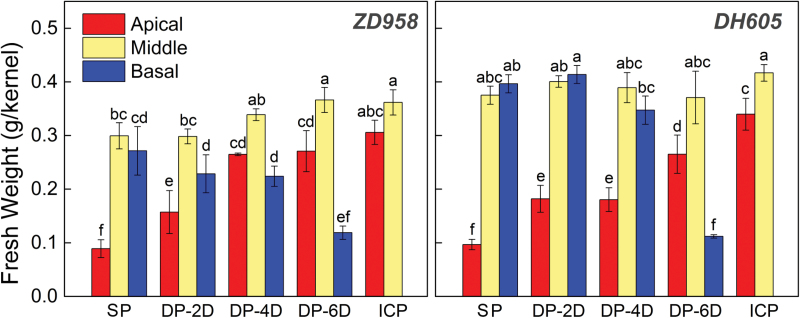
Kernel fresh weight of the maize hybrids *ZhengDan 958* (*ZD958*) and *DengHai 605* (*DH605*) at the maturity stage. Red, yellow, and blue bars represent kernels on the apical, middle, and basal area of the ear, respectively. DP-2D, pollination time for basal kernels delayed by 2 days; DP-4D, pollination time for basal kernels delayed by 4 days; DP-6D, pollination time for basal kernels delayed by 6 days; ICP, incomplete pollination treatment in which the basal silks were not pollinated; SP, synchronous pollination. One-way analysis of variance was conducted by Duncan’s new multiple range test, n=4; different letters above the bars indicate significant differences (*P*<0.05).

Some of the apical kernels in the SP treatment were aborted in the early growth stage (Fig. 4). Delaying or stopping pollination of the basal kernels in the DP and ICP treatments restored the growth of the apical kernels to different degrees. At maturity, the ICP treatment yielded the highest fresh weight of apical kernels, followed by the DP-6D, DP-4D, DP-2D, and SP treatments in order (Fig. 5). For kernels in the middle of the ear, there were no obvious differences in fresh weight across the treatments, in which all showed rapid growth (Fig. 4B, E) and developed into normal grains (Fig. 5; Supplementary Fig. S3). The fresh weight of the basal kernels in the DP treatments increased slowly compared with those in the SP treatment, and the degree of this growth retardation correlated with the length of delay of pollination (Fig. 4C, F). The basal kernels derived from the DP-2D and DP-4D treatments developed into normal seeds with slightly reduced weight at maturity (Fig. 5; Supplementary Fig. S3), whereas basal kernels from the DP-6D treatment displayed the same growth-inhibition phenotype as the aborted SP apical kernels, whose growth ceased early in development (Fig. 4C, F; Supplementary Fig. S3).

### Sugar concentrations were reduced in response to delayed pollination of basal kernels

**Fig. 6.**
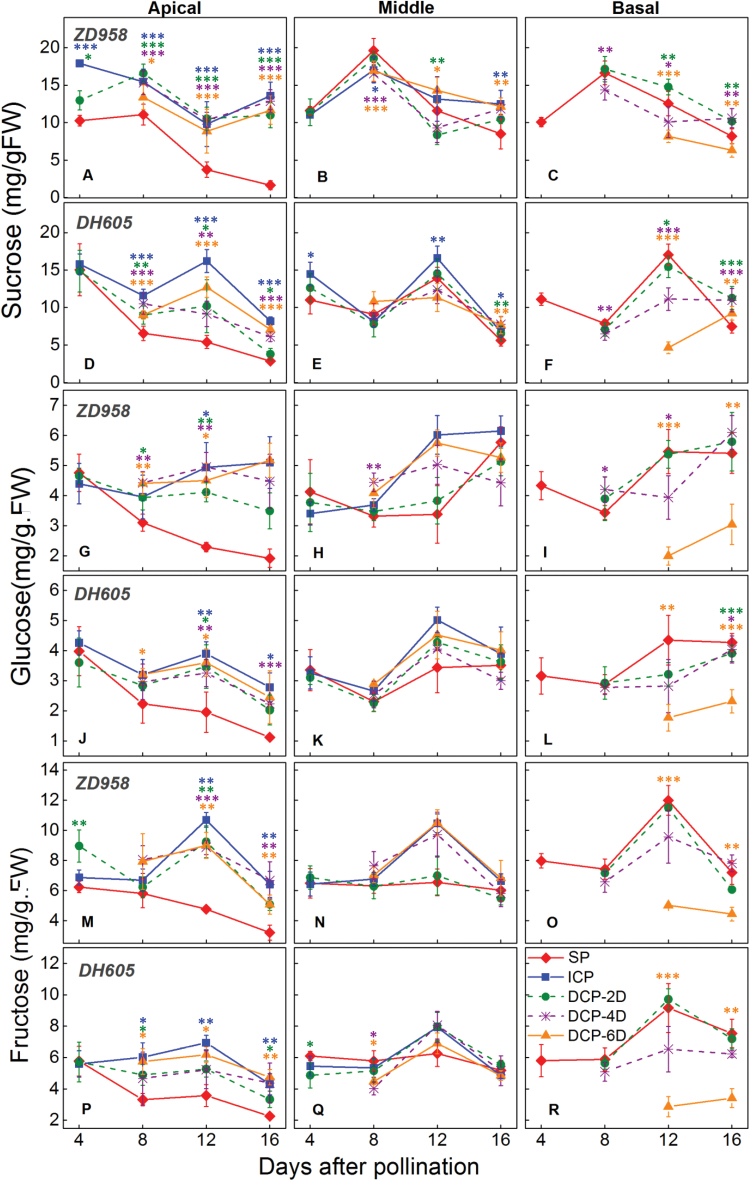
Changes in the concentrations of sucrose (A–F), glucose (G–L), and fructose (M–R) in the apical, middle, and basal kernels of the maize hybrids *ZhengDan 958* (*ZD958*) and *DengHai 605* (*DH605*) at the early kernel development stage in response to different pollination treatments. DP-2D, pollination time for basal kernels delayed by 2 days; DP-4D, pollination time for basal kernels delayed by 4 days; DP-6D, pollination time for basal kernels delayed by 6 days; ICP, incomplete pollination treatment in which the basal silks were not pollinated; SP, synchronous pollination. The concentrations of sugars are given by fresh weight (FW) of kernel tissue. Colored asterisks indicate significant differences between the SP and controlled pollination treatments (*t*-test, n=4: *P<0.05, **P<0.01, ***P<0.001).

Among apical kernels, the sucrose concentration was lowest in the SP treatment at the early growth stage (Fig. 6A, D), but was significantly increased by delayed or stopped pollination of the basal kernels in the DP and ICP treatments (Fig. 6A, D). The sucrose concentration in the middle kernels was maintained at a higher level than in the apical kernels (Fig. 6). In basal kernels, the sucrose concentration was similar in the SP, DP-2D, and DP-4D treatments for both *ZD958* and *DH605* (Fig. 6C, F), whereas in both hybrids the DP-6D treatment was associated with the lowest sucrose concentration at 12 DAP (Fig. 6F). Thus, delaying the pollination time of the basal kernels reversed the pattern in the SP treatment, in which sucrose was preferentially allocated to the basal kernels. Interestingly, the SP apical kernels and the DP-6D basal kernels were aborted with different sucrose concentrations (Fig. 6), implying that different mechanisms underlie kernel abortion in these two kernel types.

In apical kernels, hexose concentrations rapidly decreased after pollination in the SP treatment, which indicates sugar depletion in the aborted apical kernels. In contrast, hexose concentrations in the normally set apical kernels in the DP and ICP treatments increased in *ZD958* and were maintained in *DH605* (Fig. 6). In DP-6D basal kernels, the reduction in hexose concentrations was much larger than the reduction in sucrose concentrations (Fig. 6); this finding indicates that the degradation of sucrose into hexoses in basal kernels was suppressed by the DP treatments, especially by DP-6D.

### Starch quantity and localization differed in kernels exposed to different pollination treatments

**Fig. 7.**
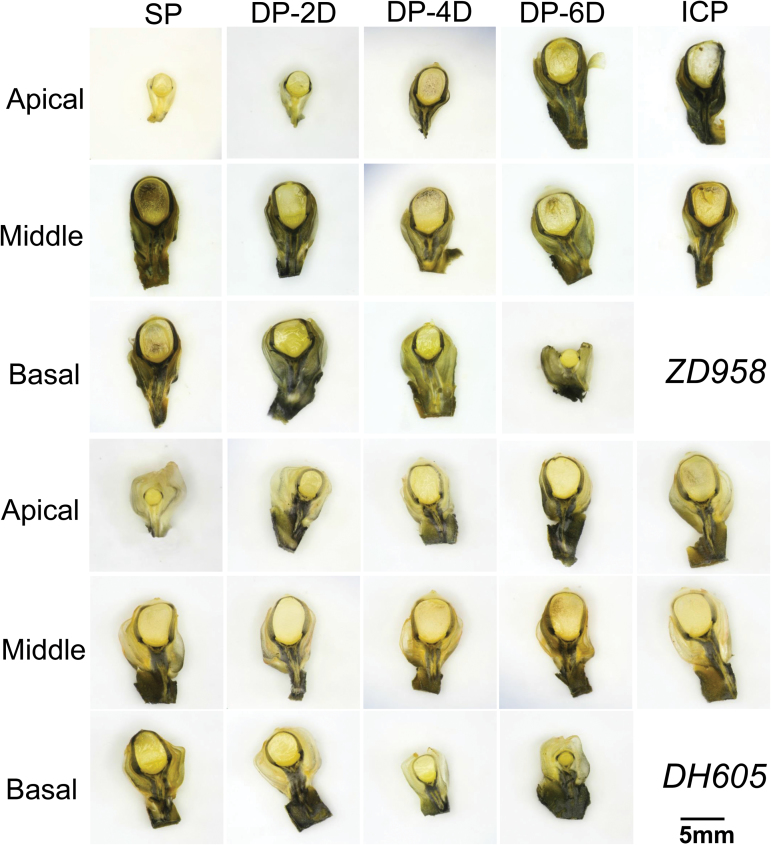
Staining of starch in sections of fresh kernels of *ZhengDan 958* (*ZD958*) and *DengHai 605* (*DH605*) hybrid maize at 8 days after pollination. Starch was located around the nucellus and vascular tissues in the pedicel. Abundant starch was present in the middle and basal kernels in all pollination treatments; in the apical kernels, starch was absent in the synchronous pollination (SP) treatment but recovered in the delayed pollination (DP) and incomplete pollination (ICP) treatments. DP-2D, pollination time for basal kernels delayed by 2 days; DP-4D, pollination time for basal kernels delayed by 4 days; DP-6D, pollination time for basal kernels delayed by 6 days. Scale bar=5 mm.

Localization analysis revealed abundant starch in the basal to middle kernels in all pollination treatments but, surprisingly, no starch in the apical kernels in the SP treatment, where the ovaries in the apical region were pollinated at the same time as those in the cob base (Fig. 7). Significantly, delayed or no pollination of the basal ovaries allowed starch to be synthesized in the apical kernels (Fig. 7). Quantification of the starch content confirmed an increase in the starch level in apical kernels and a decrease in basal kernels associated with DP of the basal kernels (Supplementary Fig. S5). These data indicate a deficient supply of assimilates to the apical kernels and a sufficient supply to the basal kernels (Fig. 7).

### Delayed pollination inhibited invertase activity

**Fig. 8.**
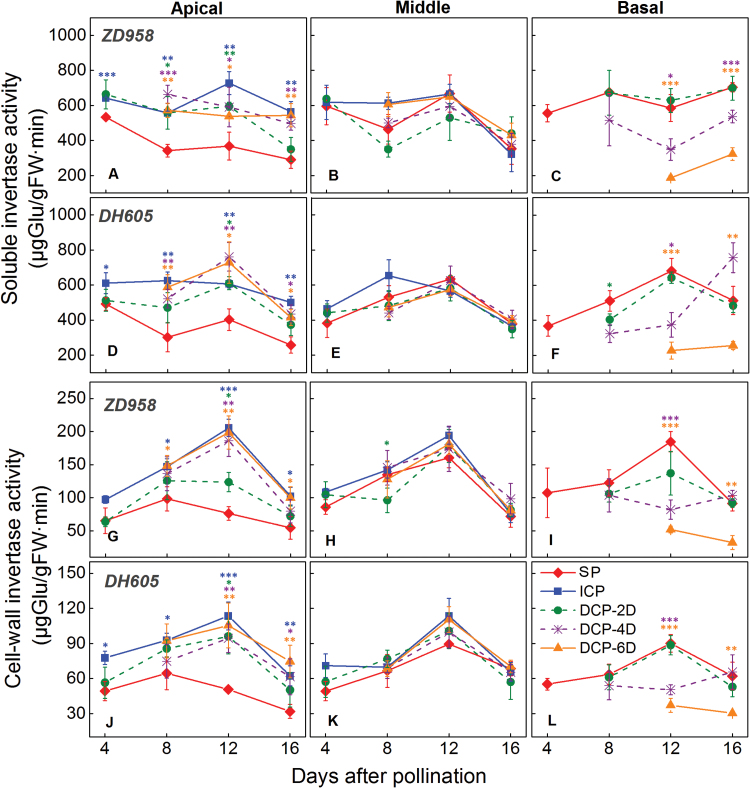
Changes in soluble invertase and cell wall invertase activity in the apical, middle and basal kernels of *ZhengDan 958* (*ZD958*) and *DengHai 605* (*DH605*) hybrid maize at the early development stage. DP-2D, pollination time for basal kernels delayed by 2 days; DP-4D, pollination time for basal kernels delayed by 4 days; DP-6D, pollination time for basal kernels delayed by 6 days; ICP, incomplete pollination treatment in which the basal silks were not pollinated; SP, synchronous pollination. The activity of the invertases is given by fresh weight (FW) of kernel tissue. Colored asterisks indicate significant differences between the SP and controlled pollination treatments (*t*-test, n=4: *P<0.05, **P<0.01, ***P<0.001).

Soluble invertase and CWIN play major roles in controlling sink strength and plant fertility (Ruan, 2014; Wan *et al.*, 2017). Enzyme assay revealed that the activities of these enzymes were altered by the different pollination treatments (Fig. 8). Invertase activities were lowest in the apical kernels from the SP treatment, and were higher to various degrees in the ICP and DP treatments (Fig. 8). In the basal kernels, the activities of the two invertases were reduced by different degrees in response to DP, with the lowest activities being detected in the DP-6D treatment (Fig. 8). This observation corresponded with a slight reduction in sucrose concentrations and a more pronounced reduction in hexose concentrations (Fig. 6). Considering that the duration of growth of DP-6D basal kernels sampled at 12 and 16 DAP were practically 6 and 12 days, respectively (i.e. if comparisons were performed according to the duration of growth instead of the sampling date), the activities of the invertases were dramatically reduced in the DP-6D treatment, whereas invertase activities in the DP-2D and DP-4D treatments were somewhat lower than those recorded in the SP treatment.

**Fig. 9.**
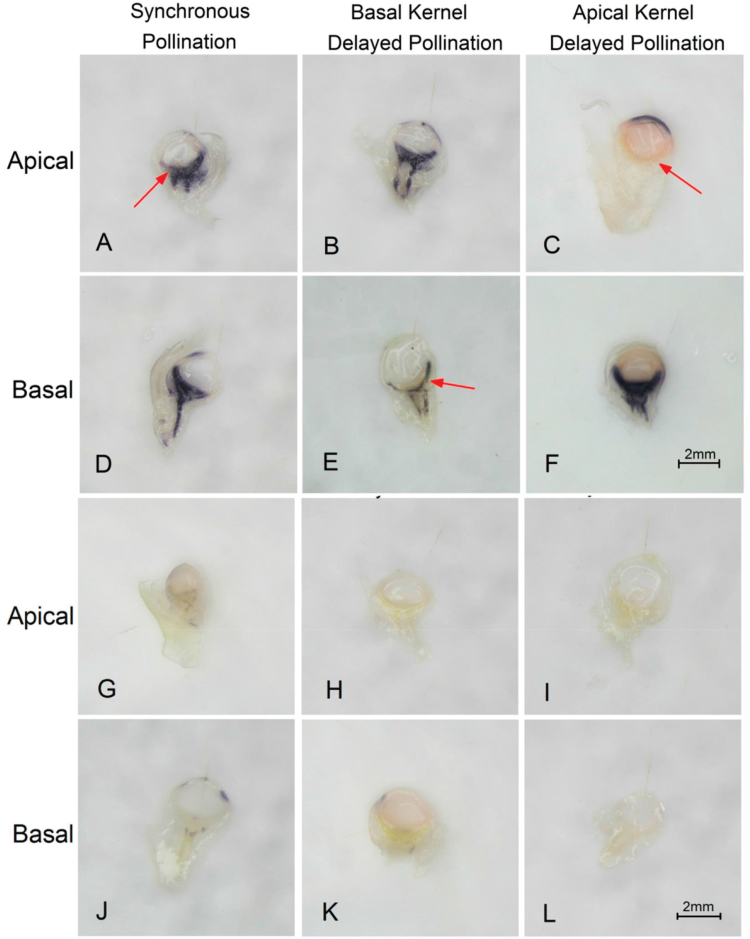
*In situ* localization of cell wall invertase (CWIN) in a fresh section of *DengHai 605* at 2 days after pollination. The CWIN is located in the upper pedicel below the spherical nucellus (the area stained black). For different pollination treatments, the apical and basal kernels were sampled separately on the second day after being hand-pollinated, when there was no obvious difference between the kernels in size and appearance. (A, D) Both apical and basal kernels showed CWIN activity when they were synchronously pollinated. (B, E) CWIN activity was detected in the normally pollinated apical kernel and 6-day-delayed pollinated basal kernel. (C, F) When the pollination of the apical kernels was delayed by 6 days relative to that of the basal kernels, CWIN activity was detected in the basal kernels, whereas, it was absent from delayed-pollinated apical kernel. (G–L) Controls of the CWIN localization assays, in which the reaction solution did not contain sucrose. Scale bar=2 mm.

To further verify this relationship between the PTGs and CWIN in the apical and basal kernels, experiment II was conducted using synchronous pollination and a 6-day delay in apical and basal pollination (Fig. 3B). In the SP treatment, strong CWIN activity was detected in both apical and basal kernels (Fig. 9A, D). However, DP of basal ovaries dramatically reduced CWIN activity in the basal kernels compared with the activity in kernels in the same position from the SP treatment (Fig. 9D, E). Application of the DP treatment to apical ovaries also reduced CWIN activity in the kernels that developed in this region (Fig. 9C) without affecting the activity in the basal region (Fig. 9F). Overall, CWIN activity was suppressed by DP independent of kernel position (Fig. 9) and was evident prior to the emergence of differences in sugar contents (Fig. 9).

### Endogenous ethylene release in aborted kernels

**Fig. 10.**
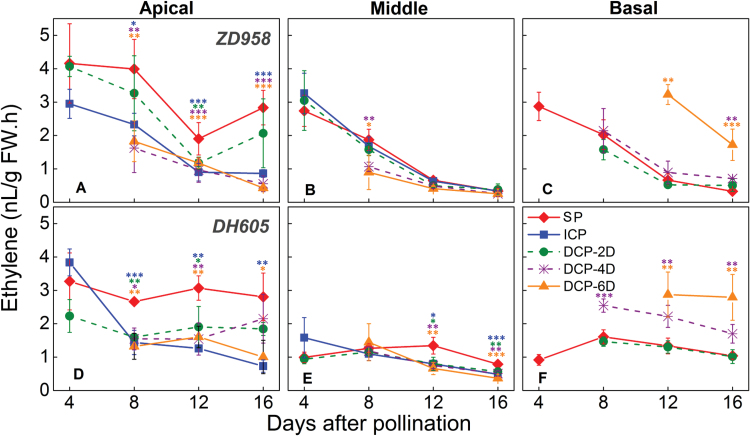
Changes in endogenous ethylene release in the apical, middle, and basal kernels of *ZhengDan 958* (*ZD958*) and *DengHai 605* (*DH605*) hybrid maize during the early kernel development period. DP-2D, pollination time for basal kernels delayed by 2 days; DP-4D, pollination time for basal kernels delayed by 4 days; DP-6D, pollination time for basal kernels delayed by 6 days; ICP, incomplete pollination treatment in which the basal silks were not pollinated; SP, synchronous pollination. Colored asterisks indicate significant differences between the SP and controlled pollination treatments (*t*-test, n=4: *P<0.05, **P<0.01, ***P<0.001).

As one of the plant hormones, ethylene has been implicated in grain abortion in cereals (Young and Gallie, 1999; Hays *et al.*, 2007). Here, we found that, in aborted SP apical and DP-6D basal kernels, ethylene levels remained at a high level early in development; in contrast, in normally set grains, ethylene emission decreased rapidly from 4 DAP and then remained at a low level (Fig. 10).

## Discussion

### Weak competition for assimilates among kernels on different ear regions is the primary driver of post-fertilized kernel abortion

From an evolutionary perspective, the advantage of overproduction of ovaries relative to the number of seeds set could be explained by (i) a ‘bet-hedging’ strategy in which plants could quickly adjust the number of seeds in response to unpredictable environments (Lloyd, 1980; Kozlowski and Stearns, 1989; Burd, 1998; Arathi, 2011) and (ii) a mechanism for eliminating ovaries of low quality and survival probability, in order to efficiently invest resources in more competitive ovaries (de Jong and Klinkhamer, 2005; Mena-Ali and Rocha, 2005; Arathi, 2011), especially when resources are deficient, for example, under abiotic stresses (Ruan, 2014; Tardieu *et al.*, 2014). This hypothesis predicts that kernel set or abortion closely relates to the amount of resources available. Consistent with this hypothesis, many previous studies have demonstrated that limitation of assimilates drives fruit and seed abortion (Reynolds *et al.*, 2009; Egli, 2010; Ruan *et al.*, 2012). However, several recent studies have suggested that sugar deprivation is a consequence rather than a cause of kernel abortion, because genes affecting expansive growth were influenced earlier than the genes affecting sugar metabolism in aborted kernels (Oury *et al.*, 2016a, b). Nevertheless, these conclusions were reached under conditions of water deficit, which could independently influence expression of genes related to kernel growth (Kakumanu *et al.*, 2012).

We showed in this study that artificially preventing basal kernel set by delaying pollination activated the apical kernels, which would otherwise have been aborted, to grow normally. We also observed that even under severe water stress, apical kernels could develop when basal kernels were repressed by blocking pollination (data not shown). These findings demonstrate the existence of competition between kernels in the basal and apical regions for the limited assimilates. Notably, the apical kernels still aborted in the SP treatment when no environmental stress was imposed (Fig. 2A, F; Supplementary Fig. S3), indicating that kernel abortion could be triggered even if pollination takes place. Collectively, these findings strongly suggest that limited availability of assimilates or resources is the driver of kernel abortion.

### Pollination time and ear region are early determinants of assimilate partitioning and selective kernel abortion

Sucrose is the major photoassimilate translocated from source to sink organs such as developing kernels. Sucrose deficiency has been proposed to be the major cause of growth suppression of maize kernels (Hanft and Jones, 1986; Boyle *et al.*, 1991; McLaughlin and Boyer, 2004*a*; Hiyane *et al.*, 2010). Consistent with this proposal, sucrose was significantly lower in aborted SP apical kernels at the early growth stage and higher in developed apical kernels following ICP to the basal ovaries. Phloem-unloaded sucrose could be used in maize kernels for synthesizing starch as a carbon reserve in the maternal tissue pedicel if it is not immediately catabolized (Zinselmeier *et al.*, 1999). Thus, starch abundance in the pedicel is indicative of the supply of assimilates to maize kernels. In this study, starch was abundant in the basal to middle kernels in all pollination treatments, whereas no starch was present in the apical kernels in the SP treatment (Fig. 7). The findings indicate that apical kernel abortion may be caused in part by sucrose depletion. Interestingly, there seems to be a certain level of sucrose available to aborted basal kernels following DP-6D treatment (Figs 6 and 7), suggesting that in addition to deficient sucrose supply, other factors may contribute to basal kernel abortion. Indeed, previous studies have demonstrated that mild water deficit can cause kernel abortion even when the sucrose supply is not depleted (Schussler and Westgate, 1995; Andersen *et al.*, 2002; Muller *et al.*, 2011). The differences between aborted and set basal kernels in glucose and fructose concentrations were more pronounced than in sucrose concentration, indicating that breakdown of sucrose into glucose and fructose was suppressed in the aborted basal kernels. Therefore, our data suggest that kernel abortion is induced by low availability of assimilates and poor ability of the kernels to cleave sucrose into hexoses.

Invertases, including CWIN and soluble invertase, hydrolyze sucrose into glucose and fructose. The invertases in sinks are particularly relevant to sucrose phloem unloading, generation of the hexose-to-sucrose ratio, sugar signaling in the fruit, and the seed set process (Koch, 2004; Ruan *et al.*, 2012; Ruan, 2014). Significantly, in this study the invertase activities were detected at low levels in aborted kernels, including the SP apical kernels and the DP-6D basal kernels (Fig. 8). The extremely low hexose and normal sucrose levels in DP-6D aborted basal kernels further point to low invertase activity as a major factor responsible for basal kernel abortion.

Invertases might control kernel growth in several ways. First, high CWIN could facilitate apoplasmic phloem unloading of sucrose to the developing grains (Ruan *et al* 2012). Second, strong CWIN activity produces adequate glucose as a signal to activate cell cycle genes, contribute to the maintenance of reactive oxygen species homeostasis, and inhibit expression of programmed cell death genes to allow seed set to proceed (Rolland *et al.*, 2006; Ruan *et al.*, 2010; Liu *et al.*, 2016). Third, CWIN may control seed growth through other signaling pathways independent of sugars (Cheng and Chourey, 1999; Muller *et al.*, 2011). Therefore, higher CWIN facilitates higher utilization of sucrose, which promotes preferential flow of the sucrose supply to, and its utilization within, developing kernels.

In species with linearly arranged ovules and kernels, the probability of abortion is often non-random with respect to position (Hossaert and Valéro, 1988; Rocha and Stephenson, 1991; Guitian, 1994; Munier-Jolain *et al.*, 1998; Jing *et al.*, 2000). The effect of seed position may involve competition for maternal resources (Watson and Casper, 1984; Rocha and Stephenson, 1991) or the timing of fertilization (Rocha and Stephenson, 1991; Cárcova and Otegui, 2001; Obeso, 2002; Uribelarrea *et al.*, 2008; Arathi, 2011; Oury *et al.*, 2016*b*). Interestingly, in maize kernel abortion preferentially occurs on the apical region of ears, which are the furthest from maternal resources, whereas abortion tends to occur on the basal end of fruits, which are the nearest to maternal resources, in cucumber (Varga and Bruinsma, 1990; Cheng *et al.*, 2015) and legumes (Hossaert and Valéro, 1988; Rocha and Stephenson, 1990, 1991; Guitian, 1994). The common feature among these species is that abortion preferentially occurs in the kernels or ovules that are pollinated later. This phenomenon may be due to a ‘head start’ effect of fertilization leading to the formation of a strong resource sink, independent of kernel position (Arathi, 2011). In this study, delaying pollination changed the partitioning of assimilates and the pattern of abortion in maize, providing strong experimental evidence for the vital role of the PTG in non-random kernel abortion and in rebalancing the trade-off between apical and basal kernel growth.

**Fig. 11.**
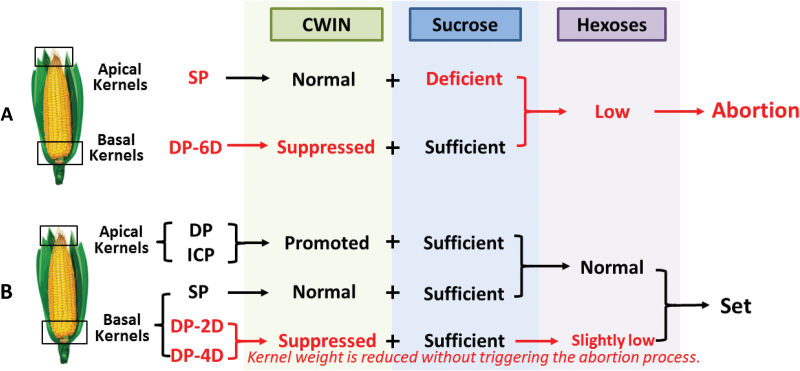
Effect of pollination time gaps on the growth of apical and basal kernels. (A) Two patterns are shown for triggering the abortion process for apical kernels exposed to synchronous pollination (SP) and basal kernels that are delayed-pollinated by 6 days (DP-6D), in which sucrose depletion and the suppression of cell wall invertase (CWIN) play separate roles. (B) Basal kernels in which pollination is delayed by 2 days (DP-2D) or 4 days (DP-4D) set normally with a slight reduction in weight, whereas DP-6D basal kernels abort, representing two different responses to delayed pollination (DP)-induced CWIN suppression. ICP, incomplete pollination treatment in which the basal silks were not pollinated.

Specifically, strong CWIN activity level was apparent in SP apical kernels (Fig. 9), which means that the abortion of SP apical kernels was not triggered by weak CWIN. However, DP of the apical and basal kernels reduced both CWIN activity and kernel set (Fig. 9), directly demonstrating the negative effect of DP on CWIN activity. The stimulating effect of the pollination process on the expression of the genes encoding CWIN has also been demonstrated in tobacco (Goetz *et al.*, 2017). Interestingly, while we observed deficient CWIN activity in the delayed-pollinated apical kernels, a certain level of CWIN activity was still present in the delayed-pollinated basal kernels. This is likely due to the fact that the basal kernels access assimilates earlier than the apical kernels, which may lead to more sucrose being unloaded into the basal kernels, activating CWIN (Roitsch *et al.*, 2003; Koch, 2004; Ruan, 2014). Thus, the PTG could block the assimilate supply by repressing CWIN activity. Since the SP apical kernels aborted with normal CWIN activity and insufficient availability of sucrose and starch (Fig. 7), we propose two different patterns for kernel abortion: deficiency in maternal resources in the apical position, and reduced or blocked sucrose delivery due to weak CWIN activity in the basal position (Fig. 11A).

### Inferior kernels respond to the competition-induced shortage of assimilates by weight reduction and abortion

**Fig. 12.**
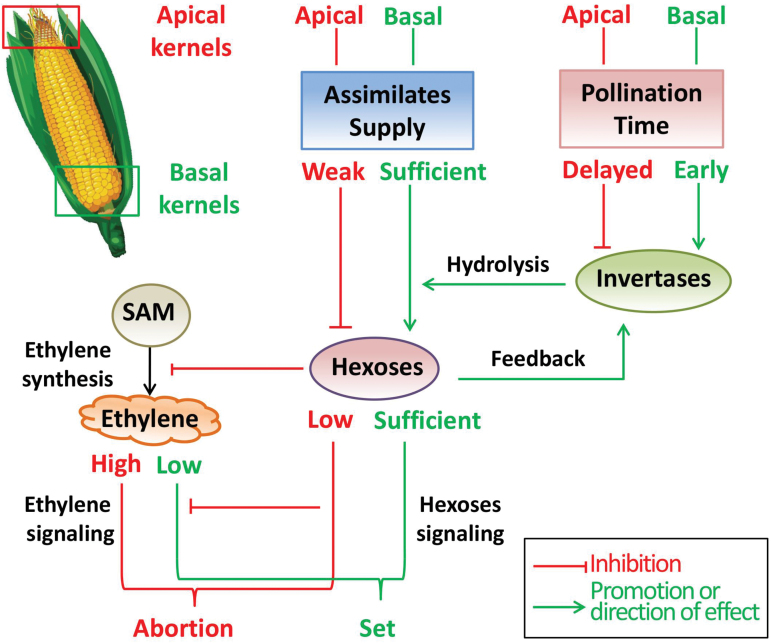
In practical maize production, the assimilate deprivation and pollination time gap-induced reduction in invertase activity both contribute to kernel abortion. Red text and arrows indicate the disadvantages for apical kernels or inhibition effects; green indicates the advantages for basal kernels or promotion and direction effects. SAM, *S*-adenosyl methionine.

In this study, we observed two different responses to DP: DP-2D and DP-4D basal kernels developed to seeds with a slight reduction in fresh weight, whereas DP-6D basal kernels stopped development and aborted (Fig. 4C, F; Supplementary Fig. S3). Weight reduction or abortion represent two phenotypes that may be under different metabolic regulatory control (Fig. 11B). Here, the switch to kernel abortion likely depends on the extent of the competition for assimilates at the most sensitive stage; when invertase activity was suppressed by DP, those kernels were inferior to normally pollinated kernels in terms of sugar metabolism. Notably, when this inferiority was intensified by increasing the PTG to 6 days, a much-reduced hexose concentration was observed in the basal kernels and the abortion process was triggered. Therefore, the mechanism by which inferior kernels switch from weight reduction to abortion may involve sugar signaling (Kakumanu *et al.*, 2012). To this end, ethylene has been suggested to be involved in ovary abortion in maize and wheat (Young *et al.*, 1997; Young and Gallie, 1999; Hays *et al.*, 2007; Cicchino *et al.*, 2013). Glucose could play a vital role in suppressing ethylene biosynthesis (Hong *et al.*, 2004; Wang *et al.*, 2014) and its signaling pathway (Yanagisawa *et al.*, 2003; Iqbal *et al.*, 2013). In this study, endogenous ethylene emission was significantly higher both in the SP apical and DP-6D basal aborted kernels (Fig. 10), which represents an opposite pattern to that observed for glucose and sucrose concentrations (Fig. 6). This observation is consistent with the view that sufficient glucose and sucrose could suppress endogenous ethylene emission (Hong *et al.*, 2004; Feng *et al.*, 2011). Thus, we suggest that a process by which sugars antagonize ethylene emission and signaling is involved in the process of kernel abortion (Fig. 12).

### Apical kernel abortion in practical maize production is determined by poor supply of assimilates and the PTG

As discussed above, we propose that a shortage of assimilates determines kernel abortion, whereas the PTG-induced decline in invertase activity further determines which kernels should be aborted, and to what extent. In practical crop production, maize kernels are pollinated naturally and asynchronously. Although the silks of the apical kernels must elongate by the smallest distance to extent out of the bracts, they are the latest-emerging silks according to the base-to-apex initiation pattern. Thus, the apical ovaries are pollinated last among all the ovaries in the cob, rendering them the weakest sinks for assimilates. Moreover, the supply of assimilates to the apical kernels is inferior to the supply to the middle and basal kernels. Owing to the concurrence of the two limiting factors, kernel abortion occurs frequently in the apical ear region in practical maize production. Future efforts should be made to alleviate these limiting factors to synchronize maize kernel development and improve grain yield.

## Supplementary data

Supplementary data are available at *JXB* online.

**Fig. S1.** Images showing the process of identifying the basal silks in a silk cluster.

**Fig. S2**. Images showing the processes used to conduct the basal kernel delayed pollination treatment.

**Fig. S3.** Ears and kernels of maize exposed to the different pollination treatments at the maturity stage.

**Fig. S4.** Images showing the results of an experiment to assess the level of silk receptiveness of *DengHai 605* (*DH605*) and *ZhengDan 958* (*ZD958*) to delayed pollination.

**Fig. S5.** Starch content in apical, middle, and basal kernels of maize hybrids *ZhengDan 958* (*ZD958*) and *DengHai 605* (*DH605*) at 8 and 12 DAP.

Supplementary Figures

## Abbreviations:

CWIN: Cell wall invertase
DP: delayed pollination
ICP: incomplete pollination
PTG: pollination time gap
SP: synchronous pollination.
