# Supplementary material for: Delayed pollination and low availability of assimilates are major factors causing maize kernel abortion
Source: J Exp Bot. 2018 Feb 9;69(7):1599–613. doi: 10.1093/jxb/ery013 (PMC5888920; doi:10.1093/jxb/ery013)
Supplement: Supplementary Figures [file ery013_suppl_supplementary_figures-s1-s5.pdf]

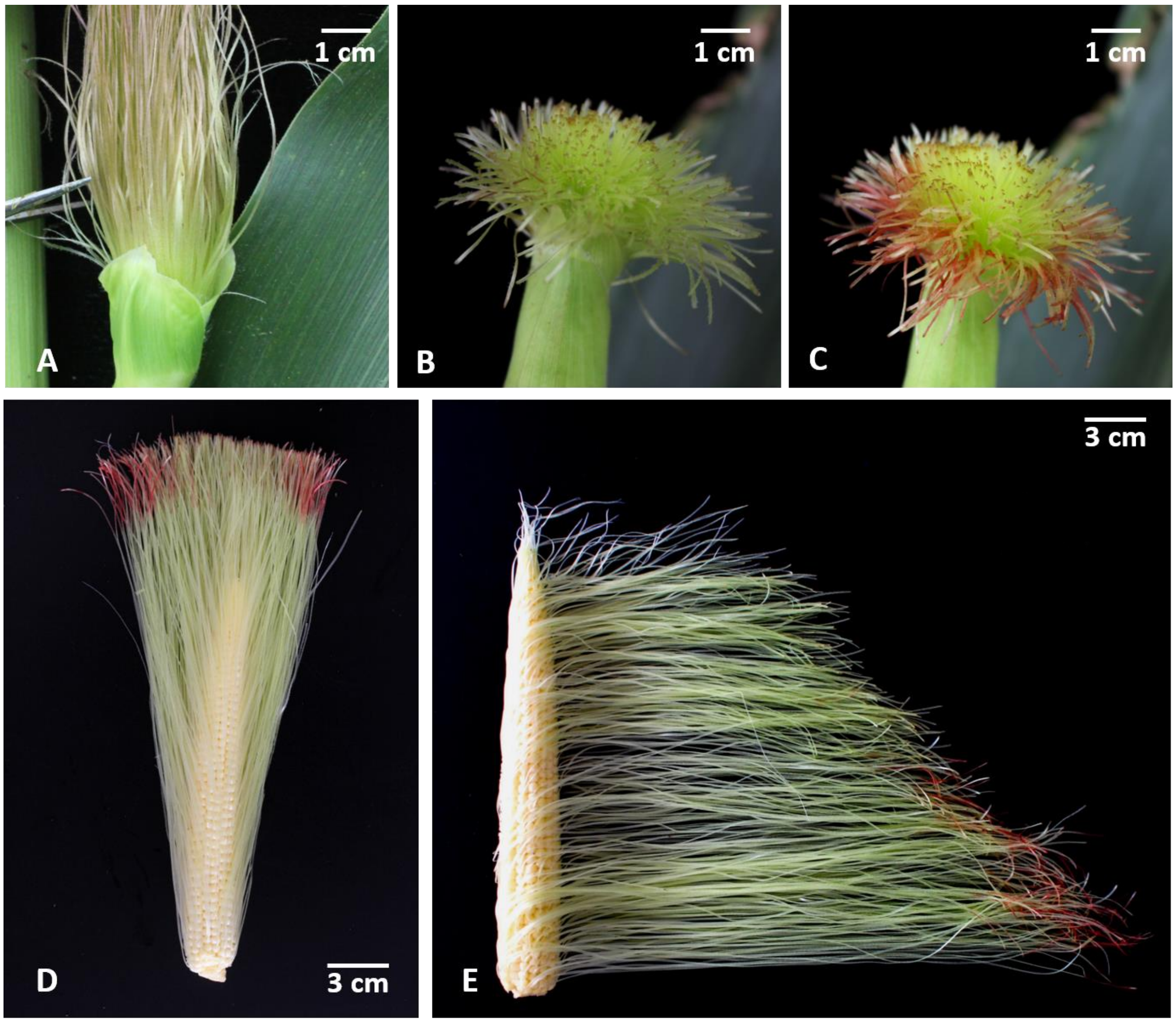

Supplemental Figure S1. Images showing the process of identifying the basal silks in a silk cluster.

(A) At 4 days after first silk emergence, all silks were fully emerged;
(B) Cut the silks at 2-3 cm from husks thus rendering them neatly arranged;
(C) Using red dyes marked silks in the outer ring and central of the silk cluster, separately;
(D) Carefully pulled off the husks without breaking the silks. Then the architecture structure of silks was observed;
(E) Each silk goes to one ovary on maize ear; the basal silks were identified to be in the outer ring of silk cluster.

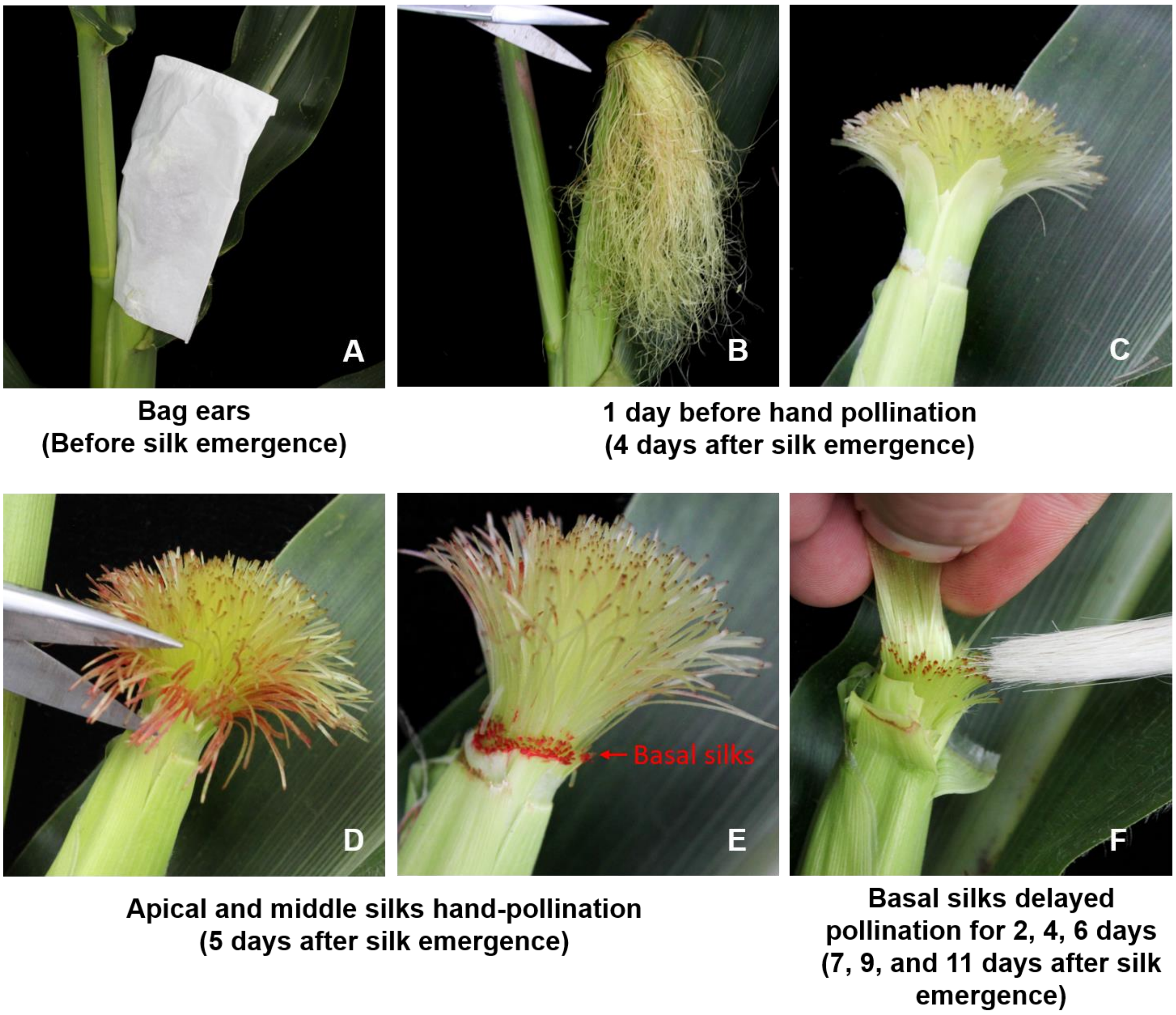

Supplemental Figure S2. Images showing the processes used to conduct the basal kernel delayed pollination treatment.

(A) Before silk emergence, using paper bags covered those selected cobs to prevent them from naturally pollination;

(B) 4 days after silk emergence, all silks fully emerged. Without pollination, silks elongated further and twist together;

(C) Cut the silks from 2-3 cm from husks to make the silks neatly arranged;

(D) Next morning, applied hand-pollination. Carefully cut those basal silks (red) along the edges of husks;

(E) Basal silks (marked by red colour) are physically hidden in husks thereby avoiding the dropped pollens. Brush fresh pollens on the rest of silks developed from the mid-apical regions;

(F) Strip husks tips and brush fresh pollens on those basal silks. Cover the ears with paper bags after every step to prevent from natural pollination.

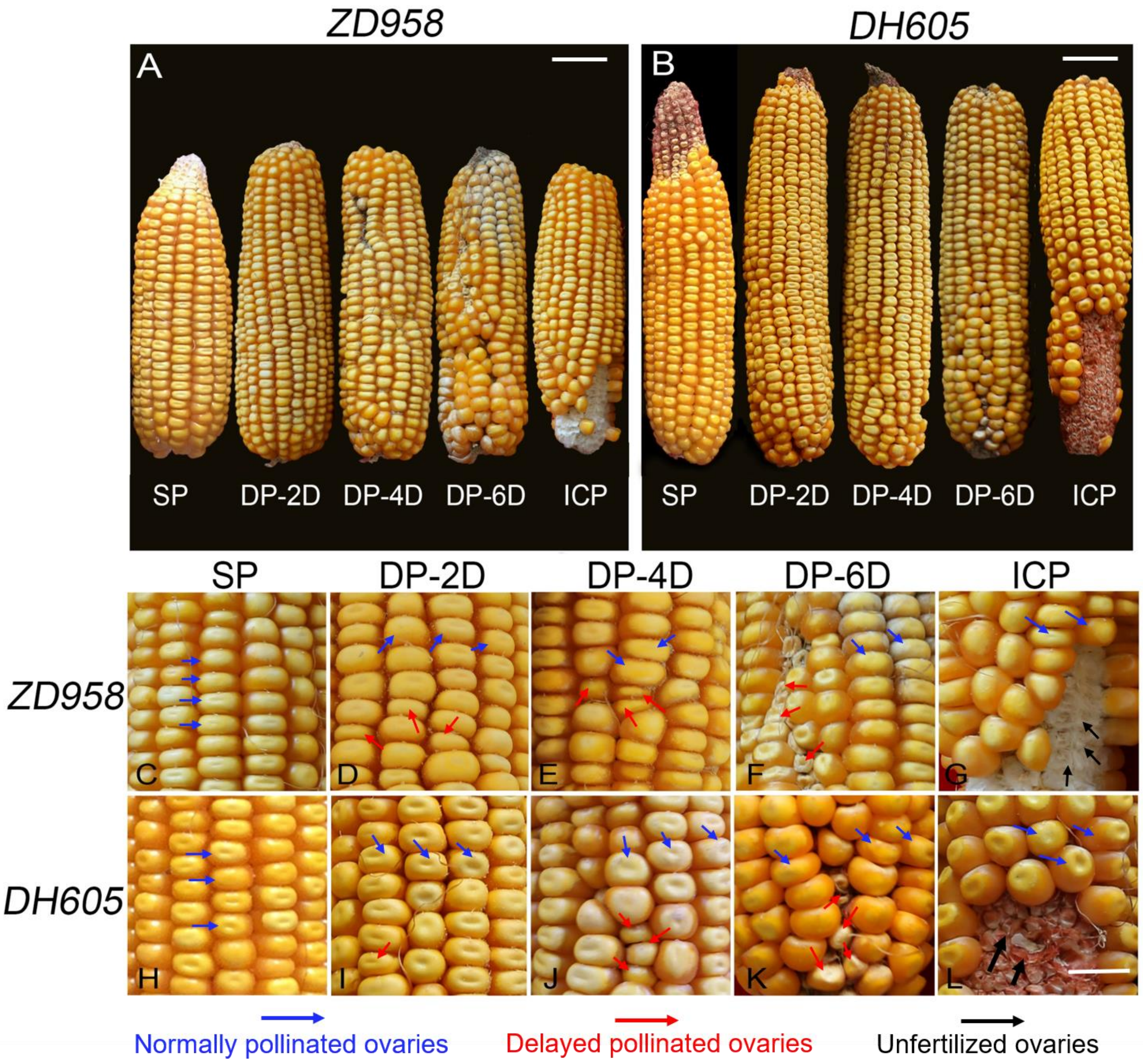

Supplemental Figure S3. Ears and kernels of maize exposed to the different pollination treatments at the maturity stage.
(A) Maize hybrid *ZhengDan 958* (*ZD958)* ears; (B) Maize hybrid *DengHai 605* (*DH605)* ears. (C-L) The magnified junction of middle kernels (normal pollination) and basal kernels (synchronous pollination in SP, delayed pollination in DP-2D, 4D, 6D and no kernels in ICP). From the junction, early pollinated and delayed pollinated kernels are easily distinguished by the colour and shape compared with SP as positive control and with ICP as negative control. The blue arrows show the middle kernels that were preferentially pollinated; the red arrows show the basal kernels that were delayed pollinated in DP treatments. SP, synchronous pollination treatment; ICP, incomplete pollination treatment in which the basal silks were non-pollinated; DP-2D, 4D, 6D, the treatments of pollination time for basal kernels was delayed 2, 4, 6 days, respectively. The scale bars are 3 cm in A and B and are 1 cm in C-L.

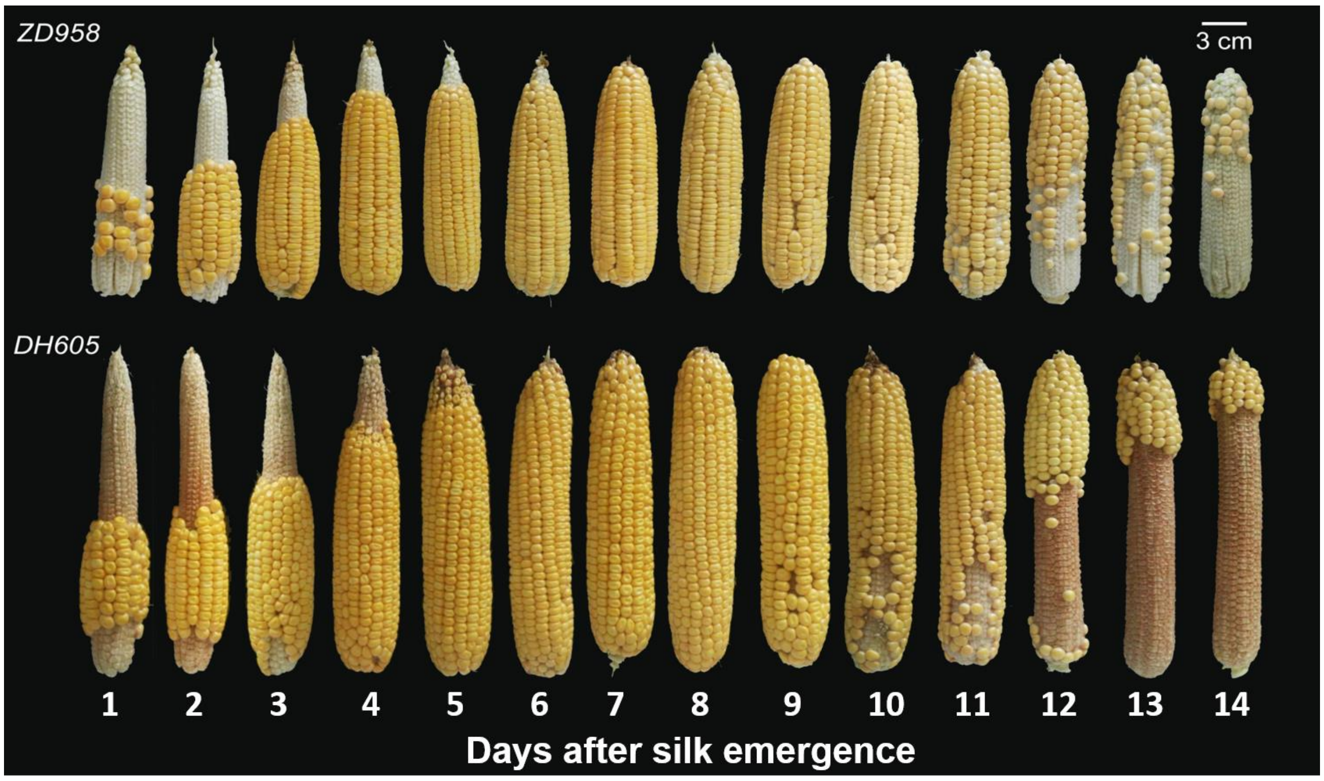

Supplemental Figure S4. Images showing the results of an experiment to assess the level of silk receptiveness of *DengHai 605* (*DH605*) and *ZhengDan 958* (*ZD958*) to delayed pollination. The emerged silks were applied with fresh pollen grains from the day of silk emergence to 14 days at 1 d interval. Before and after hand-pollination, the cobs were bagged to prevent from natural pollination. The number of fertilized basal kernels remained unaffected until 9 days after silk emergence. At least 50% of fertilized can be observed at 11 days after silk emergence, which is equivalent to delayed pollination by 6 days in this study. A majority of basal ovaries were failed to fertilization from 12 days after silk emergence.

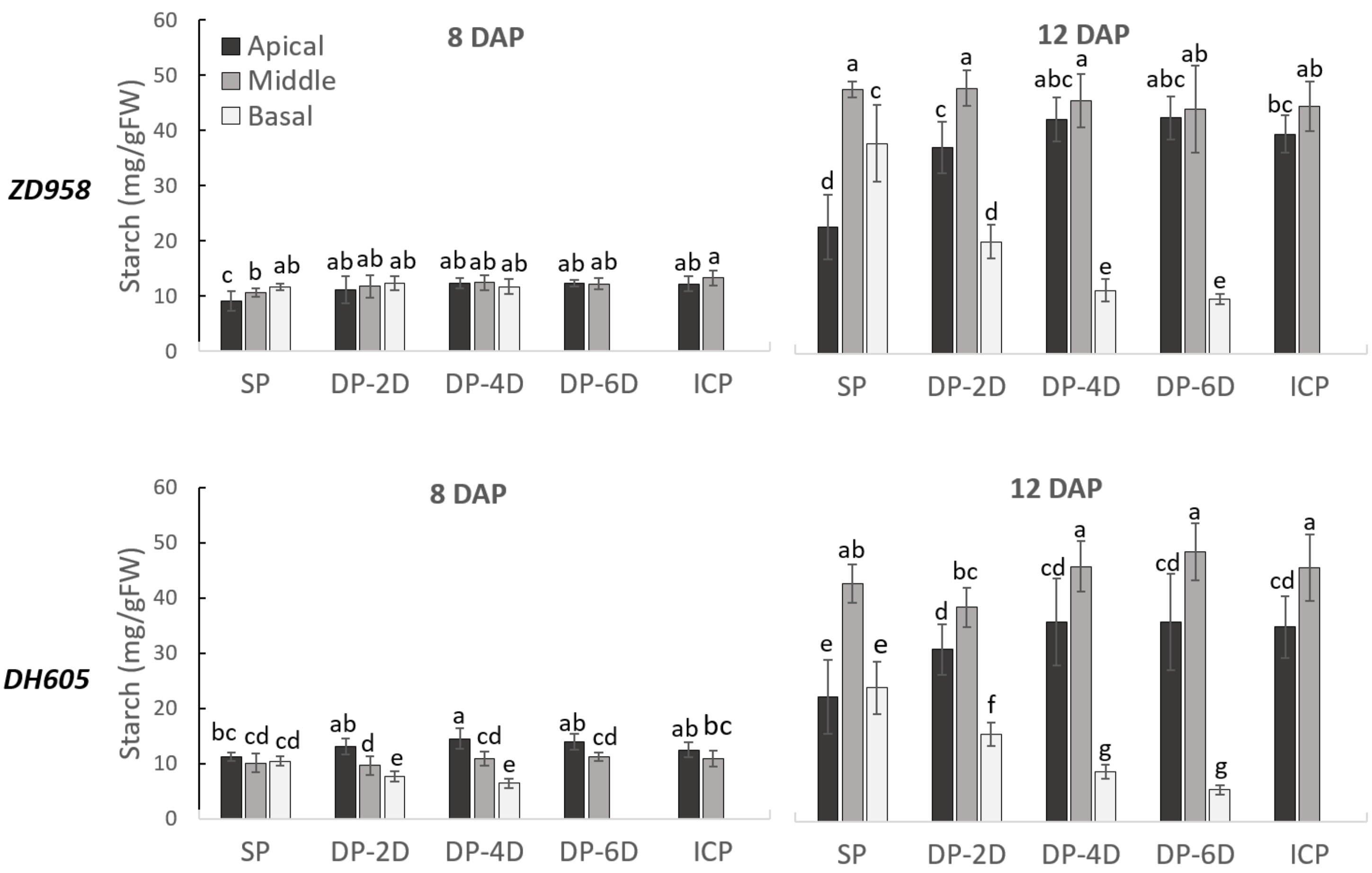

Supplemental Figure S5. Starch content in apical, middle, and basal kernels of maize hybrids *ZhengDan 958* (*ZD958*) and *DengHai* 605 (*DH605*) at 8 and 12 DAP. The kernel samples exclude the pedicel part which was showed in Fig.7. SP, synchronous pollination treatment; ICP, incomplete pollination treatment in which the basal silks were non-pollinated; DP-2D, 4D, 6D, the treatments of pollination time for basal kernels was delayed for 2, 4, 6 days, respectively. One-way analysis of variance was conducted by Duncan's new multiple range test, $P < 0.05$, n=4.
